# Editorial: Hepatic insulin resistance and related obesity: highlighting the ameliorative role of nutraceuticals, dietary intervention, and pharmaceuticals

**DOI:** 10.3389/fphar.2023.1266168

**Published:** 2023-08-07

**Authors:** Nehal M. Ramadan, Priscila O. Barbosa, Nada F. Abo El-Magd, Salma M. Eraky, Mohammed M. H. Al-Gayyar

**Affiliations:** ^1^ Clinical Pharmacology Department, Faculty of Medicine, Mansoura University, Mansoura, Egypt; ^2^ Centre for Obesity Research and Education (CORE), School of Pharmacy and Life Sciences, Robert Gordon University, Aberdeen, United Kingdom; ^3^ Department of Biochemistry, Faculty of Pharmacy, Mansoura University, Mansoura, Egypt; ^4^ Department of Pharmaceutical Chemistry, Faculty of Pharmacy, University of Tabuk, Tabuk, Saudi Arabia

**Keywords:** obesity, inflammation, drugs, natural compounds, hepatic insulin resistance, dietary intervention

*Insulin resistance* (*IR*) is the unifying denominator of all obesity-related metabolic abnormalities. It possesses a definite higher risk of developing type 2 diabetes mellitus and *non-alcoholic fatty liver disease* (*NAFLD*)*.*

NAFLD is intimately linked to an accumulation of detrimental oxidative intermediates, which in turn promote insulin receptor substrates serine/threonine phosphorylation and ultimately block hepatic insulin signalling, and *hepatic IR*. The causal relationship between hepatic IR and NAFLD is bidirectional, and hepatic IR itself is “selective” in terms of resisting only insulin’s suppressive effects on glucose production while keeping those enhancing hepatic lipogenesis intact (Ramadan et al., 2022). The present Research Topic aims to highlight the effect of natural compounds, dietary intervention, and synthetic drugs on hepatic IR and disorders related to obesity.

The selectivity of hepatic IR presents a real challenge when opting to treat obese diabetic patients with exogenous insulin as insulin can control blood glucose while the accumulation of lipids in the insulin-resistant liver induces a vicious circle that ends up in a NAFLD phenotype. In an attempt to develop a liver-targeted insulin analogue, Ardaiz et al., reported that the fusion of single-chain insulin to apolipoprotein A-I generated a novel long-acting insulin formulation selectively accumulated in the liver and, more importantly, ameliorated hepatic steatosis. Compared to recombinant human insulin, daily subcutaneous injection of the new insulin-Apo fusion protein in db/db mice efficiently improved liver histology with a significant reduction in hepatocellular lipid vacuoles.

Fenofibrate, a peroxisome proliferator-activated receptor (PPAR-α) agonist, is stated for its protective efficacy against NAFLD and associated hepatic IR which may be attributed to the alleviation of hepatic endoplasmic reticulum stress. Zhang et al. stated that the underlying molecular mechanism of fenofibrate effect could be the reduction of let-7 family miRNA expression in the livers of diet-induced NAFLD mice through upregulation of sarco/endoplasmic reticulum calcium ATPase 2b (SERCA2b), which is a target of let-7.

Fenofibrate was also the drug chosen by Whang et al., 2023, to evaluate its therapeutic effect on different non-alcoholic steatohepatitis (NASH) models in mice. The authors administered three different dosages (5 mg/kg, 25 mg/kg, and 125 mg/kg) two times a day. Treatment with 25 mg/kg of fenofibrate proved significant effect in improving hepatic steatosis, reducing fibrosis, and decreasing the number of inflammatory cells. However, a dosage of 5 mg/kg showed no significant effects. The high dose (125 mg/kg) resulted in triggered liver inflammation, increased bile acid load, and promoted liver proliferation, indicating potential toxic effects (Wang et al.). Generally, treatment with fenofibrate exhibited promising therapeutic effects in combating NASH. However, it should be noted that high doses are not always synonymous with better results.

Recently, the role of the intestinal microbiota in the development and progression of NAFLD has gained prominence in research. Changes in the composition of the microbiota and the production of short-chain fatty acids have been associated with the modulation of lipid metabolism, inflammation, oxidative stress and intestinal permeability (Ji et al., 2020). Therefore, studies involving therapeutic approaches that investigated the effects of drugs and bioactive compounds on the intestinal microbiota are crucial for more effective treatments.

In an elegant study by Li et al., the efficacy of obeticholic acid (OCA) and ferrostatin-1 (Fer-1) was tested in a mice model of obesity induced by an amylin liver NASH diet. In summary, the results of the study demonstrated that OCA, Fer-1, and their combination, offer a potential treatment strategy for the future. OCA was found to improve liver lipid accumulation, and liver fibrosis, and enrich beneficial bacteria*.* On the other hand, the effects of Fer-1 appeared to be related to weight control, glucose homeostasis, and insulin sensitivity, with an increase in bacteria from the *Lactobacillales* genus and the emergence of a new functional genus called *Blautia* **.** These findings are associated with improved glucose metabolism, reduced oxidative stress and inflammation, leading to a consequent reduction in liver injury.

In addition to studies conducted on animal models, Mohamed et al. presented promising results with cholecalciferol through a randomized controlled clinical trial. The study involved patients with NAFLD who were divided into two groups: 1) a placebo group that received conventional treatment involving physical activity and caloric restriction, and 2) a treatment group that, in addition to the conventional treatment, received a single dose of 300,000 IU of cholecalciferol followed by a daily dose of 800 IU. After 4 months of the intervention, the authors observed that treatment with cholecalciferol was able to improve lipid metabolism, serum levels of aspartate transaminase, homeostasis model assessment insulin resistance and High sensitivity C reactive protein**.** Overall, the study confirms that assessing and addressing vitamin D deficiency can be an effective approach to the treatment of NAFLD.

Furthermore, alongside the ongoing importance of discovering new drugs, bioactive compounds, including polyphenols, alkaloids, carotenoids, phytosterols, and polysaccharides, represent a natural, safer, and cost-effective approach to treat NAFLD and improve haptic IR. In this regard, de Deus et al., have provided a valuable narrative review to describe evidence supporting the antioxidant and anti-inflammatory properties of various bioactive phytochemicals; through which they probably interfere with hepatic metabolic proinflammatory signals. The effectiveness of phytochemicals in improving hepatic IR can be largely attributed to their ability to regulate NLRP3 inflammasome activation and suppress mitochondrial reactive oxygen species generation**.**

Overall, all the articles published on this Research Topic provide a comprehensive and up-to-date overview of drug therapies in animal models and a clinical trial and the role of bioactive compounds in the treatment and prevention of NAFLD through the regulation of hepatic IR pathways. These promising therapeutic approaches offer new perspectives for the clinical management of NAFLD and to prevent the complications of the disease, aiming at reducing hepatic fat, improving insulin sensitivity, decreasing inflammation and oxidative stress, as well as modulating the intestinal microbiota. As we advance in understanding the underlying mechanisms of hepatic steatosis, the use of synthetic therapies, and vitamins and taking advantage of the benefits of phytochemicals are important strategies to prevent and treat this increasingly prevalent liver disease.
